# Sustained Neurotrophin Release from Protein Nanoparticles Mediated by Matrix Metalloproteinases Induces the Alignment and Differentiation of Nerve Cells

**DOI:** 10.3390/biom9100510

**Published:** 2019-09-20

**Authors:** Yuka Matsuzaki, Rina Maruta, Keiko Takaki, Eiji Kotani, Yasuko Kato, Ryoichi Yoshimura, Yasuhisa Endo, Ciara Whitty, Christian Pernstich, Raj Gandhi, Michael Jones, Hajime Mori

**Affiliations:** 1 Department of Applied Biology, Kyoto Institute of Technology, Matsugasaki, Sakyo-ku, Kyoto 606-8585, Japan; yuka19930830@yahoo.co.jp (Y.M.); marupochi753@gmail.com (R.M.); ktakaki@kit.ac.jp (K.T.); kotani@kit.ac.jp (E.K.); ykato@kit.ac.jp (Y.K.); ryoshimu@kit.ac.jp (R.Y.); endoyasuhisa@gmail.com (Y.E.); 2 Cell Guidance Systems, Maia Building, Babraham Research Campus, Cambridge, CB22 3AT, UK; chris.pernstich@cellgs.com (C.P.); raj.gandhi@cellgs.com (R.G.)

**Keywords:** extracellular matrix, matrix metalloproteinase, neurotrophin, NGF, PC12 cells, polyhedra, protein nanoparticles

## Abstract

The spatial and temporal availability of cytokines, and the microenvironments this creates, is critical to tissue development and homeostasis. Creating concentration gradients in vitro using soluble proteins is challenging as they do not provide a self-sustainable source. To mimic the sustained cytokine secretion seen in vivo from the extracellular matrix (ECM), we encapsulated a cargo protein into insect virus-derived proteins to form nanoparticle co-crystals and studied the release of this cargo protein mediated by matrix metalloproteinase-2 (MMP-2) and MMP-8. Specifically, when nerve growth factor (NGF), a neurotrophin, was encapsulated into nanoparticles, its release was promoted by MMPs secreted by a PC12 neuronal cell line. When these NGF nanoparticles were spotted onto a cover slip to create a uniform circular field, movement and alignment of PC12 cells via their extended axons along the periphery of the NGF nanoparticle field was observed. Neural cell differentiation was confirmed by the expression of specific markers of tau, neurofilament, and GAP-43. Connections between the extended axons and the growth cones were also observed, and expression of connexin 43 was consistent with the formation of gap junctions. Extensions and connection of very fine filopodia occurred between growth cones. Our studies indicate that crystalline protein nanoparticles can be utilized to generate a highly stable cytokine gradient microenvironment that regulates the alignment and differentiation of nerve cells. This technique greatly simplifies the creation of protein concentration gradients and may lead to therapies for neuronal injuries and disease.

## 1. Introduction

The development and maintenance of the human body requires many complex interactions between cells and components of the extracellular matrix (ECM), which creates the localized microenvironments responsible for tissue development homeostasis. Control of this cellular microenvironment is therefore important for developing tissue-engineered organ substitutes. To this end, significant efforts have been directed to investigate the capacity of biomaterials and scaffolds to support and control cell growth. A number of studies have addressed topographic guidance for regulation of cell shape, orientation and movement at the interface between cells and various components of the ECM [1,2,3,4,5]. It was originally thought that MMPs primarily functioned to degrade all components of the ECM and basement membrane for tissue remodeling and maintenance. However, it has also been demonstrated that MMPs regulate the release or activation of chemokines, cytokines, growth factors, and other bioactive molecules [6]. Concentration gradients of growth factors released by MMPs are also important in tissue development, and significant efforts, for example using microfluidics [7], have been focused on the development of devices that can maintain stable concentration gradients of recombinant growth factors in vitro.

Traumatic central and peripheral nerve injury may lead to a substantial loss of nerve tissue between the proximal and the distal nerve stump. Consequently, extensive efforts have been made over the past few decades to develop treatment methods for nerve regeneration. The use of ECM mimetics, which utilize collagen gel compaction, electromagnetic fields, electrospinning of nanofibers, mechanical stimulation and microstructured culture plates for artificial guidance of nerve cells, have all been explored [8,9,10,11]. However, such artificial ECM mimetics have not led to effective therapies to address nerve injury and disease.

Previous studies have demonstrated that NGF, a member of the neurotrophin family, promotes the survival and differentiation of sensory and sympathetic neurons and may have the potential to be utilized for artificial guidance of nerve cells [12,13]. When PC12 cells, a rat neuronal precursor cell line, which has been widely used as a model for neural differentiation, are cultured in the presence of recombinant NGF, neuronal differentiation to cells that resemble sympathetic neurons, both morphologically and functionally, is observed [14]. However, the neurite outgrowth that is typically observed sprouting from the cell body lacks directionality due to the homogeneous dispersion of NGF throughout the culture medium. Molecular gradients, on the other hand, have been utilized to guide axon growth but are technically challenging to achieve and maintain. Consequently, it would be desirable to deliver NGF to the vicinity of neuronal cells by a simple, self-sustaining sustained-release mechanism that would create, over time, a similar stable microenvironment achieved by physiological supply from the ECM.

Micron-sized proteinaceous particles, known as polyhedra, are the main vectors by which virus particles transfer from insect to insect [15]. By incorporating virus particles into their crystalline superstructure, polyhedra stabilize these virions, thereby allowing them to remain viable in the external environment for long periods of time. We developed two targeted strategies for the encapsulation of foreign proteins into *Bombyx mori* cypovirus (BmCPV) polyhedra [16]. Specifically, diverse foreign proteins can be encapsulated into polyhedra by fusing a polyhedra-targeting tag sequence to the C- or N-terminus of the cargo proteins. The remarkable stability of polyhedra suggests that these systems could be robust as sustained-release carriers of cytokines and other proteins for tissue engineering or vaccination applications [16]. Moreover, polyhedrin nanoparticles are inert and insoluble in physiological conditions, allowing for polyhedra to be employed as versatile micron-sized carriers. This polyhedrin delivery system and the microscopic co-crystals it generates are both known as PODS. Here, we report the development and use of NGF-encapsulated PODS nanoparticles (pNGF), which generate slow and sustained release of NGF to direct the behavior of PC12 cells.

## 2. Materials and Methods

### 2.1. Assays for MMPs

We evaluated MMP-1, -9, and -12 (Sino Biological Inc.) and MMP-2, -3, -7, and -8 (Life Laboratory). MMP-2, -3, -7, and -8 were in their active form, whereas MMP-1, -9, and -12 required activating by chymotrypsin. MMP-1 (5 ng/μL), MMP-2, -3, and -7 (0.00025 units/μL), MMP-8 (0.00035 units/μL), MMP-9 (5 ng/μL), and MMP-12 (10 ng/μL) were added to 5 × 10^6^ pEGFP PODS in 100 μL of TCNB buffer (5 mM Tris pH 7.5, 1 mM CaCl_2_, 15 mM NaCl, 0.005% Brij-35). After incubation for 72 h at 35 °C, reactions were stopped by adding 12 μL of 0.5 M EDTA (pH 8.0). Subsequently, supernatants were collected by centrifugation and the fluorescence was measured (Ex/Em = 485/538) (Fluoroskan Ascent, Thermo Fisher Scientific, Waltham, WA, USA). The assays were carried out in triplicate.

Conditioned medium from culturing PC12 cells in various conditions was recovered and the proteins were concentrated by acetone precipitation. Subsequently, the samples were resolved by 12.5% SDS-PAGE and transferred to a nitrocellulose membrane at 2 mA/cm^2^ for 20 min. The membranes were treated with primary antibody (anti-MMP-2 antibody (Proteintech) with a 1:1000 dilution and anti-MMP-8 antibody (Boster Biological Technology) with a 1:2000 dilution) and incubated for 16 h at 4 °C. After washing three times, the membrane was incubated with a 1:2500 dilution of goat anti-rabbit IgG conjugated to horseradish peroxidase (Bio-Rad) for 2 h at room temperature. Results were visualized by Chemilumi-One (Nacalai Tesque, Kyoto, Japan).

### 2.2. RT-PCR and qPCR 

The expression of MMP-1, -2, -3, -7 and -8 mRNAs was analyzed by RT-PCR and qPCR. PC12 cells were cultured in DMEM containing pNGF (8 × 10^5^ PODS/mL) or NGF-2.5S (100 ng/mL) for 5 days. PC12 cells were also cultured in DMEM only as a control. The cDNA from cells in each culture were prepared by reverse transcription (RevertAid reverse transcriptase, Thermo Fisher Scientific, Waltham, WA, USA) from total RNA isolated using spin columns (FavorPrep^TM^, FAVORGEN, Ping-Tung, Taiwan). Products of RT-PCR were analyzed by gel electrophoresis. Relative expression of MMP genes were also determined by qPCR (CFX96, Bio-Rad) using SYBR green (Brilliant III Ultra-Fast, Agilent, Santa Clara, CA, USA). Gene expression values were given as relative expressions to the expression level in control cells. Specific primers used for qPCR are listed below. MMP-1 Forward; TTGCTTCTCTTGGCTACCAGCTCA, MMP-1 Reverse; TAGCTTGGACGTCTTCACCCAAGT, MMP-2 Forward; TGGGGGAGATTCTCACTTTG, MMP-2 Reverse; CCATCAGCGTTCCCATACTT, MMP-3 Forward; TGGGAAGCCAGTGGAAATG, MMP-3 Reverse; CCATGCAATGGGTAGGATGAG, MMP-7 Forward; TCGGCGGAGATGCTCACT, MMP-7 Reverse; TGGCAACAAACAGGAAGTTCAC, MMP-8 Forward; ACCTACGAAAATTCTACCACTTACCAA, MMP-8 Reverse; CCTTAAGCTTCTCGGCAATCA, GAPDH Forward; ACAGTCCATGCCATCACTGCC, GAPDH Reverse; GCCTGCTTCACCACCTTCTTG, Actin Forward; ATTGCTGACAGGATGCAGAA, Actin Reverse; TAGAGCCACCAATCCACACAG.

### 2.3. Construction of Expression Vectors for pNGF 

The cDNA encoding the NGF ORF was purchased from Toyobo in a GATEWAY^®^ entry clone. The full-length (241 amino acids) and mature (120 amino acids) forms of NGF were subcloned into each destination vector (pDEST/VP3, and pDEST/H1) [16], resulting in production of the transfer vectors encoding the full-length or mature form of NGF fused with VP3 or H1 tags (pTransH1/full NGF, pTransH1/mature NGF, pTransVP3/full NGF, and pTransVP3/mature NGF). These transfer vectors were co-transfected into Sf21 insect cells with BaculoGold^TM^ baculovirus linearized DNA. After incubation for 5 days at 27 °C, recombinant baculoviruses expressing the full-length and mature forms of NGF fused with VP3 or H1 tags were harvested and stored at 4 °C.

### 2.4. Expression and Purification of pNGF 

To generate polyhedra consisting of only BmCPV polyhedrin (empty PODS nanoparticles), *Spodoptera frugiperda* IPLB-SF21-AE cells (Sf cells) were inoculated with recombinant baculovirus AcCP-H29 expressing BmCPV polyhedrin under the control of the baculovirus polyhedrin promoter. For production of pNGF, Sf cells were co-infected with AcCP-H29 and an additional recombinant baculovirus expressing recombinant NGF fused with either VP3 or H1 tags [16]. The infected cells were cultured for 10 days at 27 °C and then harvested in a conical tube by centrifugation. The cell pellet was resuspended in phosphate-buffered saline (PBS; pH 7.2) and treated with an ultrasonic homogenizer at 6% power for 30 s. The cell homogenate was centrifuged at 1500× g at 4 °C and the supernatant was removed. These treatments were repeated and the purification was complete. The PODS suspension was adjusted to 5 × 10^4^ or 1 × 10^5^ PODS/μL and stored at 4 °C in distilled water containing 100 units/mL penicillin and 100 μg/mL streptomycin.

### 2.5. Cover Slip Coating 

Cover slips (ϕ 12 mm) (Fisher Scientific) were sterilized with 70% ethanol and subsequently washed with sterilized water. Cellmatrix Type IV (Nitta Gelatin) was dissolved in 0.15 M acetic acid (30 μg/mL). The sterilized cover slips were placed in the culture wells and 0.5 mL of Cellmatrix Type IV solution was then added. Following overnight incubation, the Cellmatrix Type IV solution was discarded and the cover slips were allowed to dry for 1 h. Collagen-coated cover slips were washed twice with serum-free DMEM. One microliter of PODS suspension (5 × 10^4^ or 1 × 10^5^ PODS/μL) was spotted on the cover slips and allowed to dry for at least 3 h.

### 2.6. PC12 Cell Culture 

PC12 cells (RCB0009 RIKEN BRC) were cultured with DMEM culture medium supplemented with 10% fetal bovine serum and 10% horse serum. The cell culture medium was discarded and 0.02% EDTA solution was added. After the EDTA solution was discarded, 1 mL of 0.25% trypsin-EDTA was added and incubated for 1 min. One milliliter of DMEM culture medium was added and the cell suspension was centrifuged at 1200 rpm for 2 min. After discarding the supernatant, cells were suspended in 1 mL of serum-free DMEM and the number of cells was counted. Cells (7 × 10^4^ cells/well) were then seeded into a well and incubated with serum-free DMEM medium for 5 days without medium exchange. Images of cell alignment were obtained via scanning electron microscopy (SEM) on the 5th day following cell seeding. The NGF 2.5S subunit (NGF-2.5S) from murine submaxillary glands (Sigma Aldrich, Darmstadt, Germany) was also used as a control.

### 2.7. Preparation of Cells for SEM Imaging

After gently discarding the cell medium, cells were fixed with 4% paraformaldehyde phosphate buffer solution, 1% osmium tetroxide and 1% tannic. Dehydration was carried out by immersing the cover slips in a series of ethanol solutions of increasing concentrations until 100% dehydration was achieved. Cover slips were covered with hexamethyldisilazane and allowed to dry overnight. Images of cell alignment were obtained via SEM after gold-sputtering (200 Å).

### 2.8. Immunocytochemistry

For immunofluorescence, cells on the cover slips were fixed for 30 min at room temp with 4% paraformaldehyde. After three washes with PBS for 5 min each, the cells were permeabilized with 0.3% Triton X-100 in PBS for 15 min at room temperature. After three washes with PBS for 5 min each, the cells were incubated in blocking buffer (3% FBS in PBS) for 1 h at room temperature and then incubated with primary antibodies (anti-tau antibody (Merck Millipore, Darmstadt, Germany), anti-neurofilament heavy polypeptide antibody (Sigma Aldrich, Darmstadt, Germany), anti-GAP-43 antibody (Merck Millipore, Darmstadt, Germany), and anti-connexin 43 antibody (Invitrogen, Thermo Fisher Scientific, Waltham, WA, USA) overnight at 4 °C. After three washes with PBS for 10 min each, the cells were incubated with FITC-conjugated secondary antibody (goat anti-mouse IgG antibody (Invitrogen) for 1 h at room temperature. Cover slips were then washed three times with PBS and finally mounted on microscope slides in mounting medium with propidium iodide (Invitrogen) for nuclei staining. Stained cells were observed using an Olympus Fluoview FV1000-IX81 confocal microscope (Olympus, Tokyo, Japan).

## 3. Results

### 3.1. Release of Cargo Proteins from PODS Crystals

We have previously reported that biologically-active cytokines are sustainably released from PODS nanoparticles, when the nanoparticles are co-incubated with mammalian cells or conditioned medium [17,18]. However, the mechanism by which release is mediated had not been elucidated. 

We hypothesized that the degradation of the nanoparticles is achieved by proteases, specifically MMPs, secreted by nearby PC12 cells. MMPs are proteolytic enzymes belonging to the zinc protease superfamily that has several sub-groups. These include collagenase (MMP-1, -8, -13), gelatinase (MMP-2, -9), stromelysin (MMP-3, -10, -11), matrilysin (MMP-7), and other membrane associated MMPs [19]. The MMP family is involved in the breakdown of ECM in normal physiological processes, such as embryonic development, reproduction, and tissue remodeling. MMPs are secreted as inactive pro-proteins but are activated when cleaved by extracellular proteinases [19,20]. 

**Figure 1:**
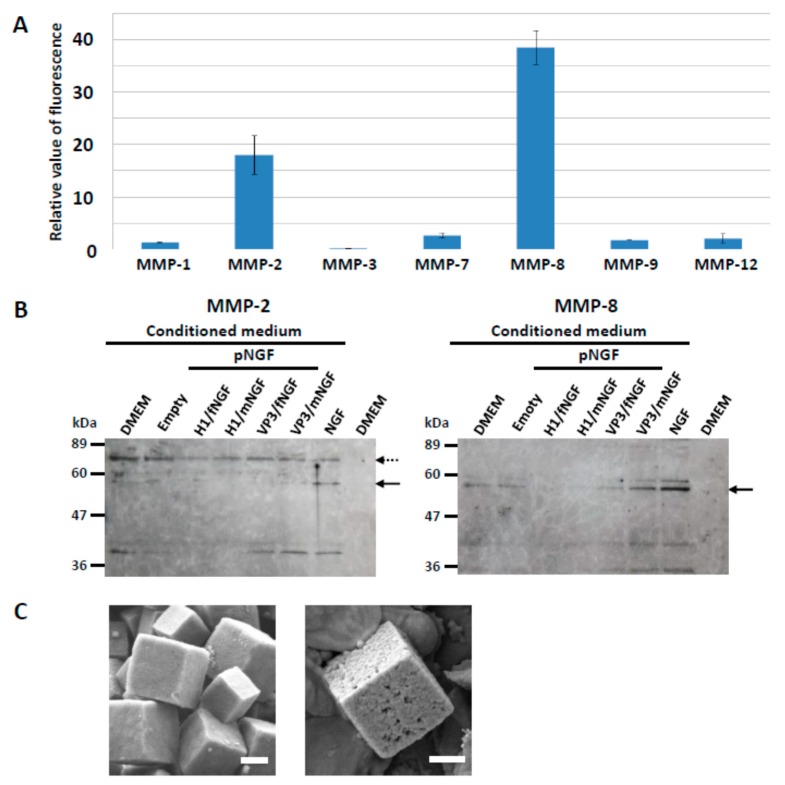
Release of cargo proteins by MMPs. (**A**) Analysis of proteins released from PODS. After H1-tagged pEGFP was incubated with MMP-1, -2, -3, -7, -8, -9, and -12 for 72 h at 35 °C, the fluorescence of EGFP released from nanoparticles was measured and shown as a relative value compared with a condition without each MMP. The relative fluorescence value was obtained by comparison with controls which were incubated without enzyme in Supplementary Table S1. (**B**) Secretion of MMPs from PC12 cells. Cells were incubated in serum free medium (DMEM) containing pNGF (NGF full-length (fNGF) and mature (mNGF) tagged with either H1 or VP3), empty PODS nanoparticles (Empty) and NGF-2.5S (NGF). Proteins secreted into the medium (conditioned medium) were subjected to SDS-PAGE and Western blot analysis by use of anti-MMP-2 (left) and anti-MMP-8 (right) antibody. The arrow and hatched arrow indicate the active form and pro-form, respectively. The larger protein fragment (seen above the band indicated by the arrow in the MMP-8 panel) is likely derived from a single nucleotide polymorphism of the MMP-8 gene [21]. Lower bands were also thought to be the degraded products of each MMP. (**C**) PODS nanoparticles were purified (left, control) and subsequently incubated with proteases at 37 °C (right) and imaged with scanning electron microscopy (SEM). Scale bars, 1 µm.

To investigate whether selected MMPs from across several sub-groups do indeed degrade PODS nanoparticles, enhanced green fluorescent protein (EGFP) was incorporated into PODS nanoparticles (pEGFP) [16], and incubated for 72 h with activated recombinant MMP-1, -2, -3, -7, -8, -9, and -12. We subsequently measured the resulting fluorescence (Figure 1A, Supplementary Table S1). Significant release of intact fluorescent EGFP was observed with MMP-2 and MMP-8 but none of the other MMPs tested appeared to promote the release of intact, fluorescent EGFP. However, further experiments showed that substantial amounts of EGFP itself were degraded following incubation with two of the seven MMPs, MMP-3 and MMP-7, and it is therefore unknown whether these MMPs are able to degrade PODS nanopraticles (Supplementary Figure S1, Table S2). MMP-2 and MMP-8 are classified as collagenases and gelatinases, respectively. Both enzymes, which are present in the connective tissue of most mammals, are known to be cleaving enzymes for tissues. These results suggest that these MMPs can be used for release of a cargo protein from PODS nanoparticles. On the other hand, when pEGFP was incubated with trypsin or chymotrypsin, significant release of intact EGFP was not observed. The reason was thought to be dependent on the presence of many sites in the EGFP molecule that are cleaved by trypsin and chymotrypsin (Supplementary Table S3). We have concluded that an environment containing proteinases with a relatively high specificity for polyhedrin is required to obtain the release of intact, biologically active cargo proteins from PODS nanoparticles. We observed the change of the surface of PODS nanoparticles incubated with proteases (Figure 1C). It was confirmed that many pores are formed on the surface of PODS nanoparticles following protease treatment.

**Figure 2:**
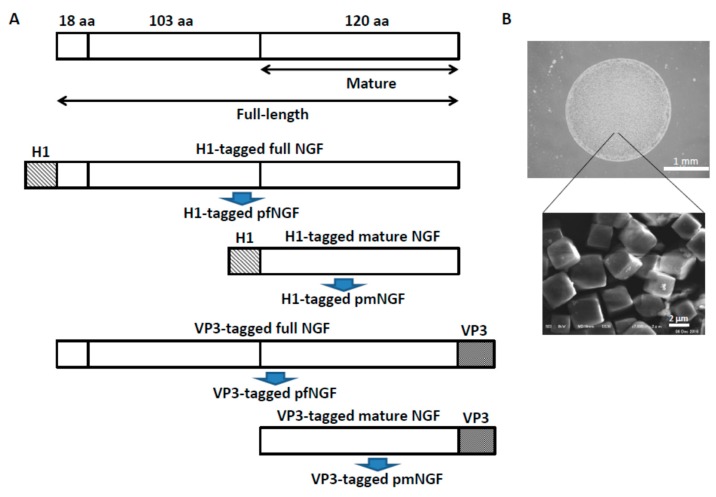
Construction and spotting of NGF-encapsulated protein nanoparticles. (**A**) Full-length and mature NGF (fNGF and mNGF, respectively) were fused with either a H1 or VP3 tag, and each recombinant NGF fusion protein was encapsulated into polyhedra to make H1-tagged or VP3-tagged pfNGF and H1-tagged or VP3-tagged pmNGF, respectively. (**B**) A spot of pNGF and SEM image of pNGF.

In order to confirm that MMPs from PC12 cells are a likely source of proteases that degrade PODS protein nanoparticles, the expression of MMPs by PC12 cells was studied using western blot analysis, reverse transcription PCR (RT-PCR), and quantitative PCR (qPCR). Full-length NGF (fNGF) or mature NGF (mNGF) was fused with polyhedra-targeting tags (H1 or VP3) to obtain NGF-encapsulated PODS nanoparticles (pfNGF or pmNGF) (Figure 2A). PC12 cells were incubated in serum-free medium containing pfNGF and pmNGF tagged with either H1 or VP3), empty PODS nanoparticles and NGF-2.5S.

Western blot analysis of the culture media showed that both MMP-2 and MMP-8 were indeed secreted into the medium by PC12 cells: both proteins could be detected in conditioned media across all culture conditions but not in non-conditioned medium (Figure 1B). We noted that MMP-2 and MMP-8 were secreted at similar levels to PC12 cells cultured with empty PODS nanoparticles and with medium only (Figure 1B), suggesting that secretion was not stimulated by either the polyhedrin protein or the released cargo protein NGF. This observation was also confirmed by investigating the levels of gene expression of MMPs in PC12 cells using RT-PCR and qPCR (Supplementary Figures S2 and S3). Although MMP-1 is produced by fibroblasts in a variety of connective tissues [19] and MMP-7 is mainly expressed in epithelial cells in the endometrium, small intestine, breast, parotid, pancreas, liver, prostate, dermis, and bronchus [22], the expression of MMP-1 and MMP-7 in PC 12 cells was not detected by qPCR analysis. It is known that the expression of MMP-3 is induced by NGF [23,24], and this phenomenon was confirmed for NGF-2.5S by qPCR (Supplementary Figure S3). However, activation of MMP-3 expression was not observed following incubation with pNGF. The expression of MMP-3 may be related to the difference in differentiations of PC12 cells incubated with NGF-2.5S and pNGF as shown below.

### 3.2. Alignment of PC12 Cells by pNGF

**Figure 3:**
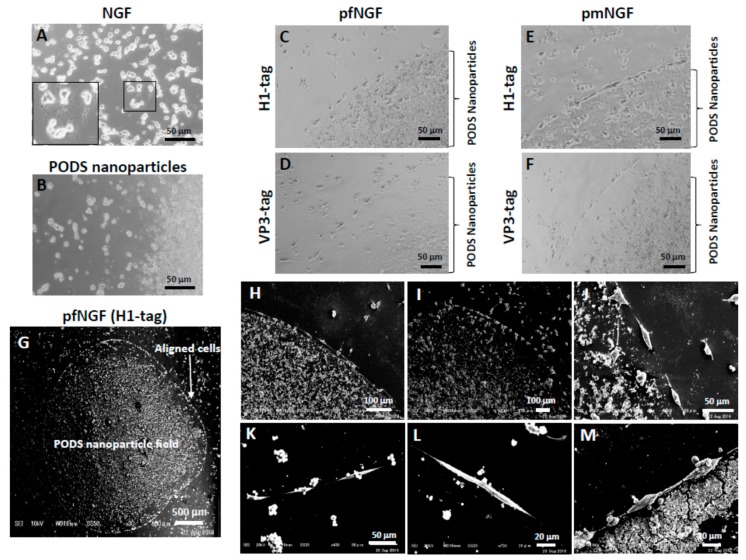
Alignment of PC12 cells surrounding the periphery of a PODS nanoparticle field. PC12 cells were seeded and incubated with NGF-2.5S from the murine submaxillary gland (NGF) (**A**). The box is a magnified image to show randomly extended axons. After empty PODS nanoparticles (**B**) and PODS NGF of H1-tagged pfNGF (**C**,**G**,**H**,**J**), H1-tagged pmNGF (**E**,**I**), VP3-tagged pfNGF (**D**,**K**,**L**,**M**), and VP3-tagged pmNGF (**F**) were spotted onto collagen-coated cover slips. PC12 cells were seeded and incubated for 5 days without medium exchange.

One microliter of either a high or low concentration PODS nanoparticle suspension of either pfNGF or pmNGF (5 × 10^4^ or 1 × 10^5^ PODS/μL) was manually spotted onto a gelatin-coated coverslip using a micropipette to create a circular field approximately 2–4 mm in diameter (Figure 2B). PC12 cells were seeded across the coverslip and cultured with serum-free medium under static conditions for 5 days without any media changes. Both pfNGF and pmNGF prominently induced axon extension from each cell (Figure 3C–F). Surprisingly, this axon extension was parallel with the edge of the PODS crystal field resulting in the formation of a connected chain of migrated cells (Figure 3G–M). In contrast, when PC12 cells were incubated with the conventional NGF-2.5S, axons were randomly extended from cells (Figure 3A). Furthermore, extension of axons was not observed for empty PODS crystals, i.e., without encapsulated NGF (Figure 3B). Alignment and extension of axons from PC12 cells surrounding pfNGF and pmNGF fields was also observed using scanning electron microscopy (SEM) (Figure 3G–M). 

To determine the extent of the gradient, pfNGF were mixed with pEGFP, and subsequently spotted on a cover slip. PC12 cells were then seeded and the fluorescence emission on the periphery of the aligned PC12 cells was measured (Supplementary Figure S4). A gradient of green fluorescence was observed from the PODS nanoparticle field, suggesting the formation of NGF concentration gradients resulting in concentrations of NGF that are critical or adequate to induce differentiation and alignment of PC12 cells. We hypothesize that the mechanism that results in such cell alignments is as follows. Firstly, MMPs are secreted by PC12 cells. NGF was released from the pNGF field by these MMPs. An imbalance and asymmetry of NGF concentration forms due to the localization of pNGF. Finally, the PC12 cells aligned and axons were extended at an area of maximum concentration in the NGF field.

### 3.3. Differentiation of PC12 Cells 

**Figure 4:**
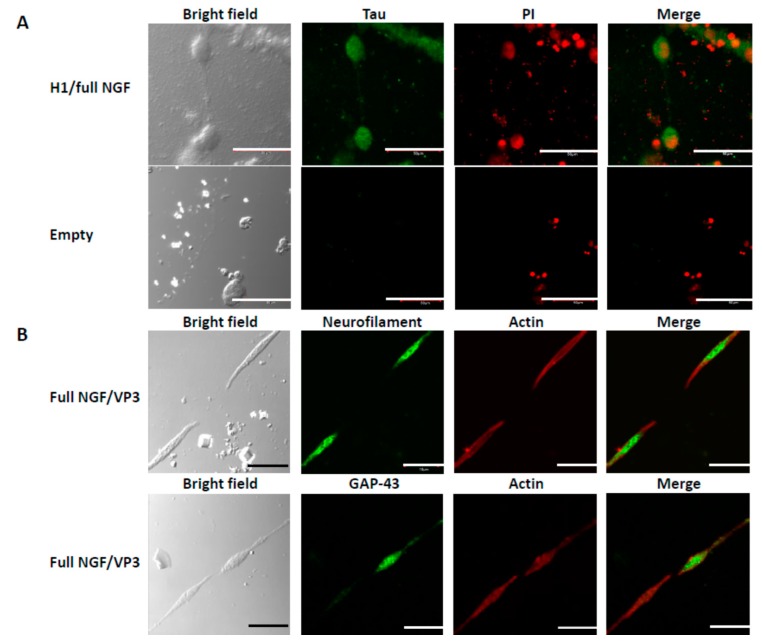
Expression of neural differential markers in the aligned PC12 cells. (**A**) Expression of tau. PC12 cells were incubated with H1-tagged pfNGF (H1/full NGF) or empty PODS nanoparticles (Empty). Expression of tau and nuclei were detected by immunofluorescence cytochemistry and propidium iodide (PI) staining. (**B**) Expression of neurofilament, GAP-43 and actin. PC12 cells were incubated with VP3-tagged pfNGF (Full NGF/VP3). Each protein was detected by immunofluorescence cytochemistry. Scale bars, 50 μm.

We examined expression of tau, neurofilament, and GAP-43, which are neural differentiation markers (Figure 4). These differentiation markers were detected in the PC12 cells aligned by the pfNGF, but no signal was observed from PC12 cells incubated adjacent to empty PODS crystals (Figure 4A). Notably, an intermediate filament protein neurofilament was also detected in the extended axon, indicating that the aligned cells were differentiated to nerve cells. It is known that NGF induces not only the formation of growth cones in PC12 cells, but also another kind of neurite terminal structure, the varicone, which has a hybrid character of growth cone and varicosity [14]. Expression of GAP-43 was observed in some of the neurite termini of the extended axons from cells facing each other but not in the neurite termini of all such cells. It is known that GAP-43 is concentrated in axonal growth cones, but is not detected in growing dendrites and dendritic growth cones, suggesting that anisotropy exerts an effect on the extension of neuronal differentiation with various types of growth cones formed by differentiation of PC12 cells caused by pfNGF.

### 3.4. Connections Between Cells

**Figure 5:**
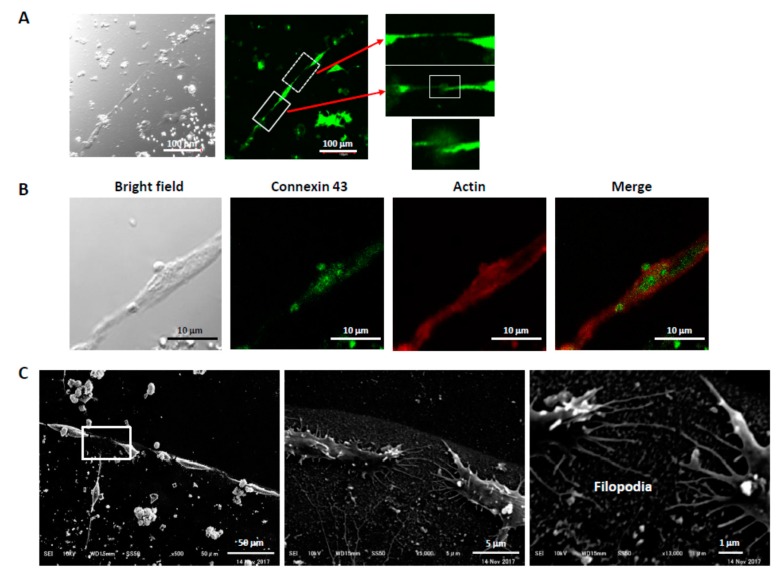
Connections between the aligned PC12 cells. (**A**) Connection via the extended axon. The extended axon induced by H1-tagged pfNGF was detected by expression of neurofilament. Connections between the extended axon and the growth cone-like structure (solid box) and between the axons (dotted box) were observed. (**B**) Expression of connexin 43. A gap junction protein, connexin 43, was detected in connecting sections of the extended axons. (**C**) Connection by filopodia. Very fine filopodia were extended and connected between growth cones.

In some cases, connections of the PC12 cells aligned by pfNGF were observed between the extended axon of one PC12 cell and the growth cone-like structure of an adjacent PC12 cell (solid box in Figure 5A). In other cases, the connection between PC-12 cells was less clear from neurofilament staining (dotted box in Figure 5A). Nonetheless, formation of connections between the neurite terminals was confirmed by the expression of a gap junction protein. Expression of connexin 43, a gap junction protein, was observed in connecting sections of the neurite terminals from aligned cells (Figure 5B). Additionally, extension of very fine filopodia from growth cones was observed by SEM and, interestingly, filopodia facing each other appear to be connected (Figure 5C). These results showed that pfNGF induced many types of structures on the neurite terminals of the extended axons from the aligned PC12 cells and also suggested a possibility of synaptogenesis including the formation of electrical synapse between the aligned cells.

## 4. Discussion

Recombinant cytokines are conventionally added to cell culture medium to promote cell differentiation; however, they rapidly diffuse and become homogenously distributed in the medium. This limits the utility of recombinant cytokines as their imbalance and asymmetry is important for the formation of tissue and organs. The in vitro generation of a gradient (or other concentration imbalance) that is physiologically relevant has been technically challenging. In addition to maintaining that gradient for a sufficiently long period of time to affect cell behavior, the gradient has to be finely tuned. Metrics such as gradient incline (which may be too steep or too shallow), starting concentration (too high or too low) and uniformity are complex parameters that can all be expected to have an impact on effect. We showed here that complex gradient phenomena that allow in vivo neuronal alignment could be simply reproduced in vitro utilizing a static field of PODS.

NGF is an important neurotrophic factor responsible for the growth, differentiation, and survival of sympathetic and neural crest-derived sensory neurons. Although PC12 cells are immortalized and do not always behave in the same way as primary neuronal cells, stimulation of pheochromocytoma PC12 cells by NGF leads to growth arrest and neuronal differentiation. In the presence of NGF, PC12 cells also differentiate into sympathetic-like neurons [25]. Furthermore, NGF promotes microtubule assembly thereby regulating neurite formation during neuronal differentiation [26], and NGF-treated PC12 cells exhibit many of the hallmarks of differentiated neurons. These characteristics combine to make PC12 cells a useful model for studying neuronal differentiation and neurite outgrowth.

In our cell cultures, we observed PC12 cells forming a single chain around the pNGF field with a distinct zone on the periphery of this region, which is devoid of cells. We also showed that pNGF induced differentiation to neural cells with a variety of neurite terminals and connections via axons, growth cones, and filopodia. These axonal connections were also perpendicular to the gradient allowing the PC12 cells to form a single, unbranched chain. We speculate that the PC12 cells in the chain are producing an inhibitor that prevents additional PC12 cells from migrating up along the NGF gradient and joining the structure.

PODS nanoparticles have the ability to release proteins over long periods of time, but are eventually degraded. This is important for therapeutic applications. In our previous study [27], bone morphogenetic protein-2 (BMP-2)-encapsulated nanoparticles (pBMP-2) were implanted with an absorbable collagen sponge (ACS) in critical-sized bone defects. This resulted in near-complete bone healing at 15 weeks following implantation. Many PODS remained intact with the ACS 5 weeks after implantation, but were not visible, being entirely replaced with newly generated bone at 15 weeks. Although we tested the in vivo implantation of three other PODS nanoparticles in addition to pBMP-2 [28,29,30], there was no evidence of inflammatory or foreign-body reaction from the host tissues adjacent to the implantation of PODS nanoparticles.

It is important to understand the mechanism by which PODS release their cargo. In this study, we showed that the EGFP cargo protein in PODS was released by MMP-2 and MMP-8. As it was confirmed that PC12 cells expressed and secreted MMP-2 and MMP-8, we conclude that the gradient of NGF was formed consequent to the secretion of these proteases by PC12 cells. 

Release of intact cargo protein requires degradation of surrounding polyhedrin protein. This process will be more efficient if the cargo protein is more resistant to MMPs. This may be expected given the different origins of the proteins. Not withstanding their typically short half-lives, growth factors have evolved to function in the metalloprotease-rich environment of the ECM. In contrast, polyhedrin protein has evolved to maintain integrity in external environments (such as foliage surfaces) that do not require resistance to MMPs. Therefore, polyhedrin may be inherently more susceptible to MMPs, enabling intact release of MMP-resistant cargo proteins and functionality of the PODS system.

NGF promotes neurite outgrowth. When NGF-2.5S is homogenously distributed in the medium, PC12 cells randomly extended axons (Figure 3A). However, when localized NGF PODS crystal fields were created, the appearance of PC12 cells was radically altered compared with appearance in the presence of soluble NGF-2.5S (Figure 3G). It has been shown previously that proteins are slowly released from PODS [18]. The resulting microenvironment resulted in the induction of differentiation and alignment of PC12 cells. We conclude that the differences were dependent on the anisotropic stimulation of PC12 cells by NGF. Although it is difficult to control the optimum concentration of NGF for nerve cell differentiation and alignment via use of an artificial ECM, pNGF may allow the creation of a gradient that faithfully mimics the in vivo ECM in which cytokines are retained and secreted, and contribute to nerve regeneration and the construction of nerve cell networks.

When a gap is less than 5 mm, a common strategy for peripheral nerve injuries is to join the distal and proximal stumps of the damaged nerves by microsurgery. When the gap is longer than 5 mm, direct microsurgery generates tension in the nerve fibers. Therefore, a nerve graft is required to fill the gap and make the connections between the distal and proximal stumps of the damaged nerves. We have already shown that neural cells are aligned along the periphery of a pNGF field with a diameter of about 2 to 5 mm (Figure 2B and Figure 3G). We estimate that our current methods will allow for the generation of about 6 to 15 mm nerve fibers for nerve grafts into an injured nerve. Although the side effects of the implantation of PODS nanoparticles were not observed in our previous study [27,28,29,30], further experiments will be needed to elucidate utility for the repair or regeneration of nerve fibers in vivo.

## 5. Conclusions

We have proposed a model wherein MMPs secreted by mammalian cells break down PODS nanoparticles and enable the sustained release of intact cargo proteins. The bioactivity of released proteins will be dependent, in part, on their susceptibility to proteases. In this experiment, NGF encapsulated into PODS nanoparticles was slowly released by MMP-2 and MMP-8 secreted from PC12 cells. Consequently, anisotropic stimulation by the released NGF induced both differentiation and alignment of PC12 cells.

## Supplementary Materials

The following are available online at https://www.mdpi.com/2218-273X/9/10/510/s1, Figure S1. Effects of MMPs on EGFP, Figure S2. RT-PCR of MMP-2, -3, and -8 expression, Figure S3. Relative qPCR of MMP-2, -3, and -8 expression, Figure S3. Gradient of the encapsulated proteins released from polyhedra. Table S1. Fluorescence of EGFP released from crystals after incubation with MMPs, Table S2. Fluorescence of soluble EGFP after incubation with MMPs, Table S3. Fluorescence of EGFP after incubation with trypsin and chymotrypsin.
