# Supplementary material for: Sustained Neurotrophin Release from Protein Nanoparticles Mediated by Matrix Metalloproteinases Induces the Alignment and Differentiation of Nerve Cells
Source: Biomolecules. 2019 Sep 20;9(10):510. doi: 10.3390/biom9100510 (PMC6843502; doi:10.3390/biom9100510)
Supplement: Supplementary file 1 [file biomolecules-09-00510-s001.zip › Supplementary Data/Supplementary Figure S1.pdf]

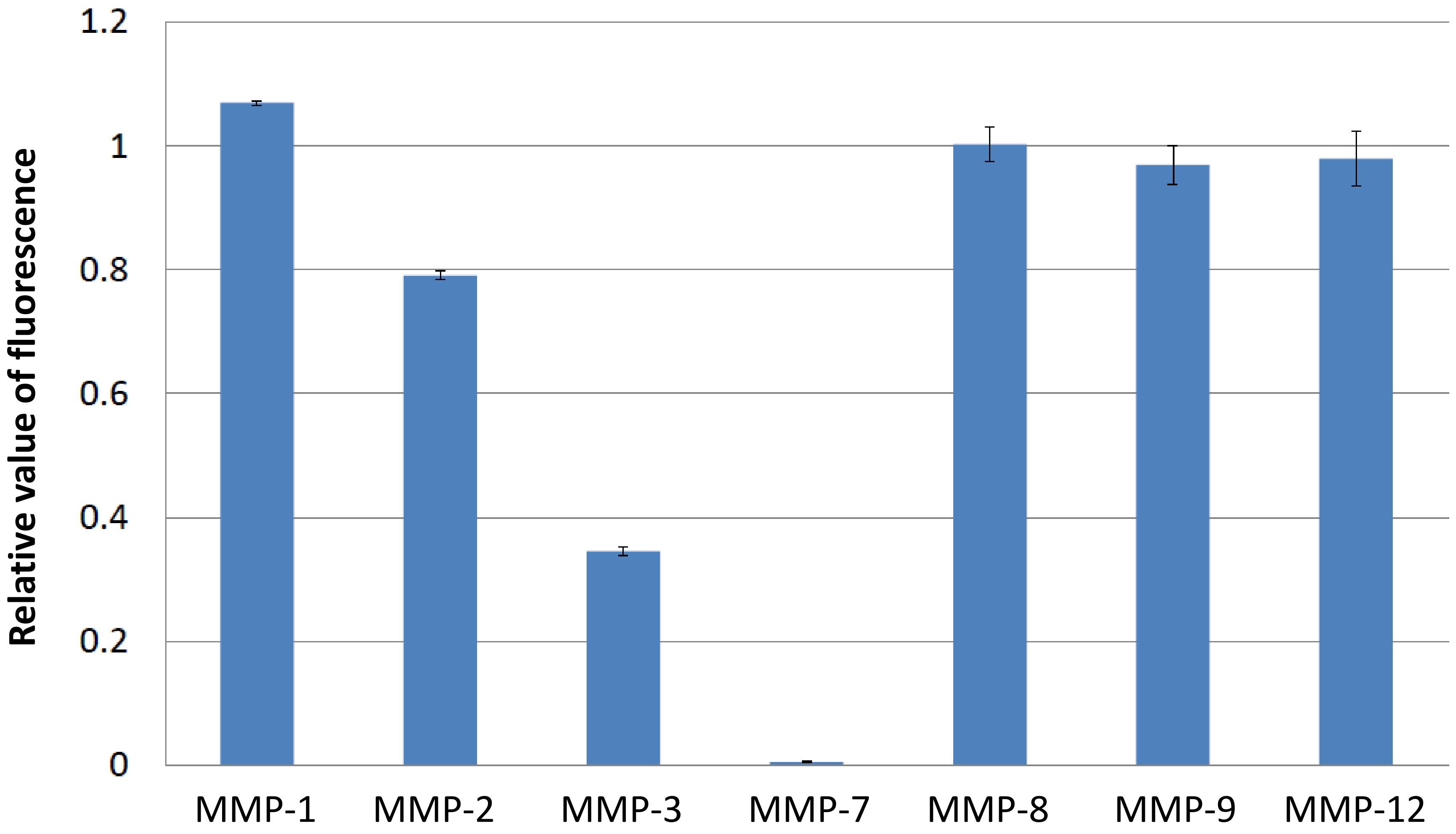

Supplementary Figure S1. Effects of MMPs on EGFP. Recombinant EGFP was incubated with each MMP as indicated in 100 µl of TCNB buffer (5 mM Tris pH 7.5, 1 mM $CaCl_2$, 15 mM NaCl, 0.005% Brij-35). After incubation at 35° C for 72 h, reactions were stopped by 12 µl of 0.5M EDTA (pH8.0) and supernatants were collected by centrifugation. Subsequently, fluorescence was measured in a plate reader (Ex/Em=485/538). Resulting values are plotted, normalized against a condition without each MMP (i.e. mock digest) set to 1.
