# Supplementary material for: Sustained Neurotrophin Release from Protein Nanoparticles Mediated by Matrix Metalloproteinases Induces the Alignment and Differentiation of Nerve Cells
Source: Biomolecules. 2019 Sep 20;9(10):510. doi: 10.3390/biom9100510 (PMC6843502; doi:10.3390/biom9100510)
Supplement: Supplementary file 1 [file biomolecules-09-00510-s001.zip › Supplementary Data/Supplementary Figure S2.pdf]

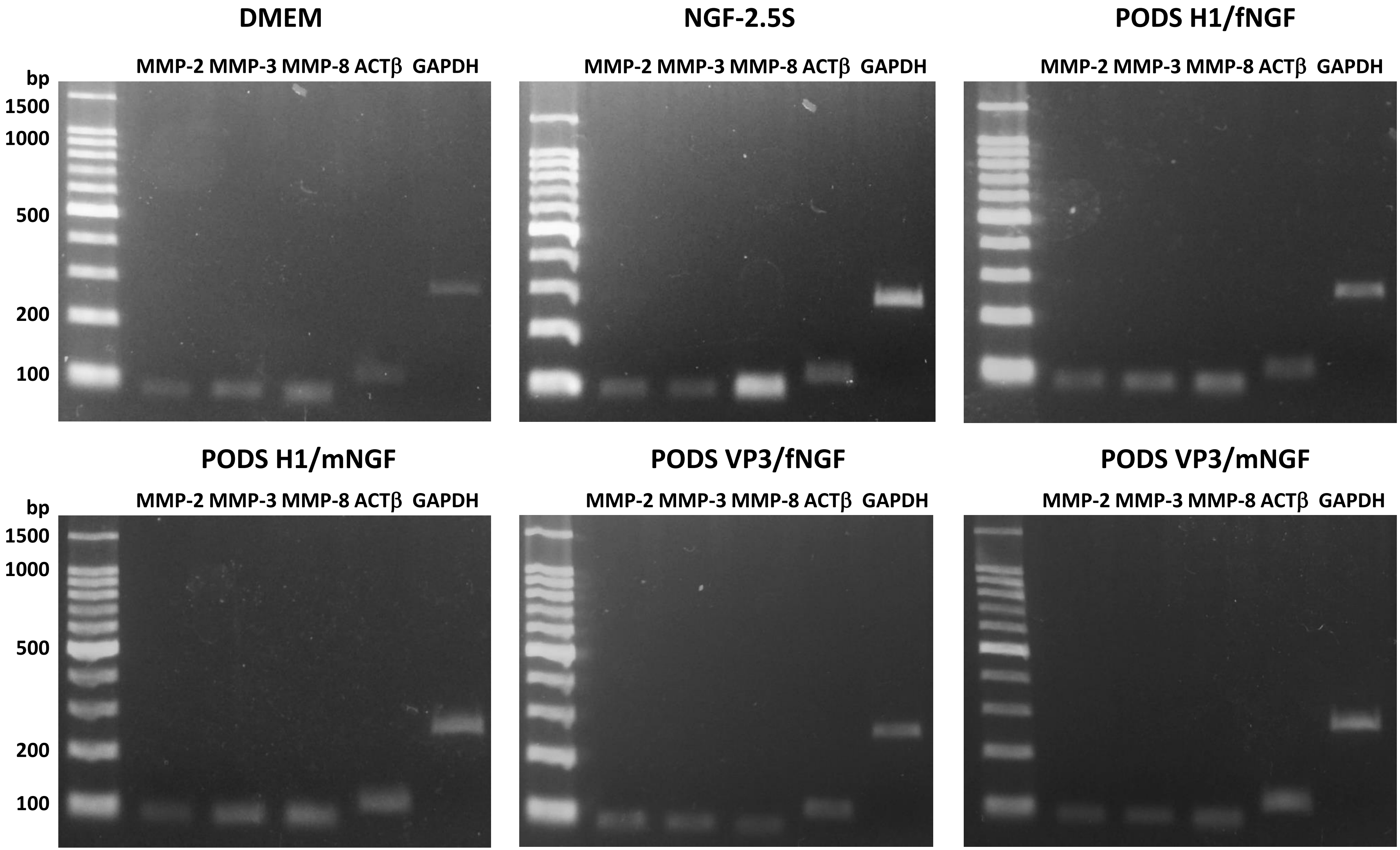

Supplementary Figure S2. RT-PCR of MMP-2, -3, and -8 expression. RCR amplifications were performed by each number of cycles (34 for MMP-2, 36 for MMP-3, 40 for MMP-8). Housekeeping genes**,** actin β (ACTβ) and glyceraldehyde-3-phosphate dehydrogenase (GAPDH) were also amplified by 22 cycles.
