# Supplementary material for: Sustained Neurotrophin Release from Protein Nanoparticles Mediated by Matrix Metalloproteinases Induces the Alignment and Differentiation of Nerve Cells
Source: Biomolecules. 2019 Sep 20;9(10):510. doi: 10.3390/biom9100510 (PMC6843502; doi:10.3390/biom9100510)
Supplement: Supplementary file 1 [file biomolecules-09-00510-s001.zip › Supplementary Data/Supplementary Figure S3.pdf]

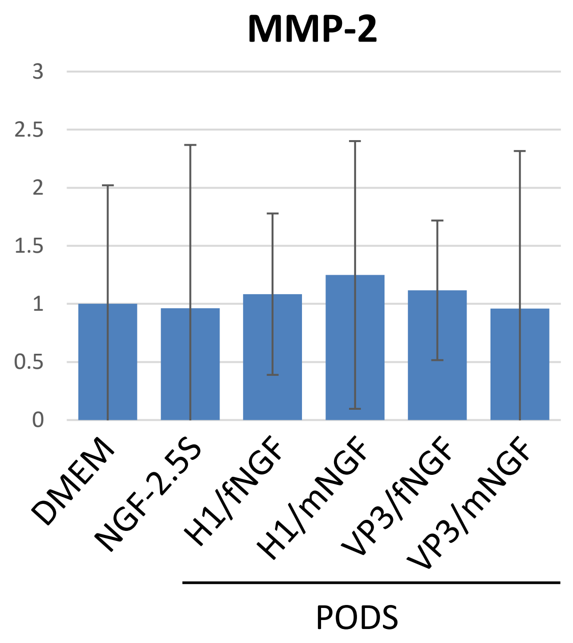

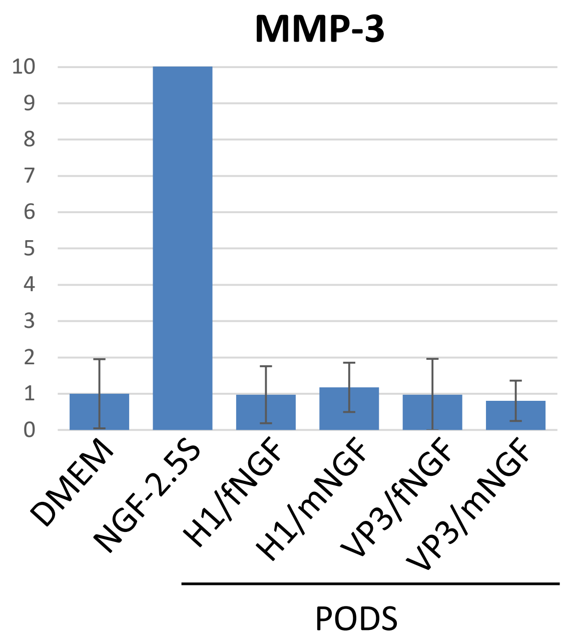

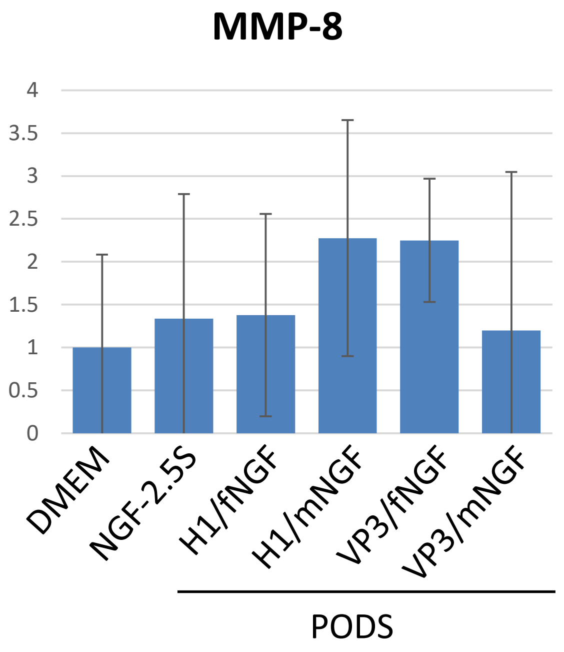

Supplementary Figure S3. Relative qPCR of MMP-2, -3, and -8 expression. Expressions of MMP-2, -3, and -8 were normalized by expression of housekeeping genes, ACTβ or GAPDH and then were shown as a relative value compared with the expression of each MMP in a culture with serum-free DMEM. There was no significant difference in a mode of the expressions of MMP-2, -3, and -8 between actinβ and GAPDH. These results normalized by the expression of GAPDH are shown.
