# Supplementary material for: Sustained Neurotrophin Release from Protein Nanoparticles Mediated by Matrix Metalloproteinases Induces the Alignment and Differentiation of Nerve Cells
Source: Biomolecules. 2019 Sep 20;9(10):510. doi: 10.3390/biom9100510 (PMC6843502; doi:10.3390/biom9100510)
Supplement: Supplementary file 1 [file biomolecules-09-00510-s001.zip › Supplementary Data/Supplementary Figure S4.pdf]

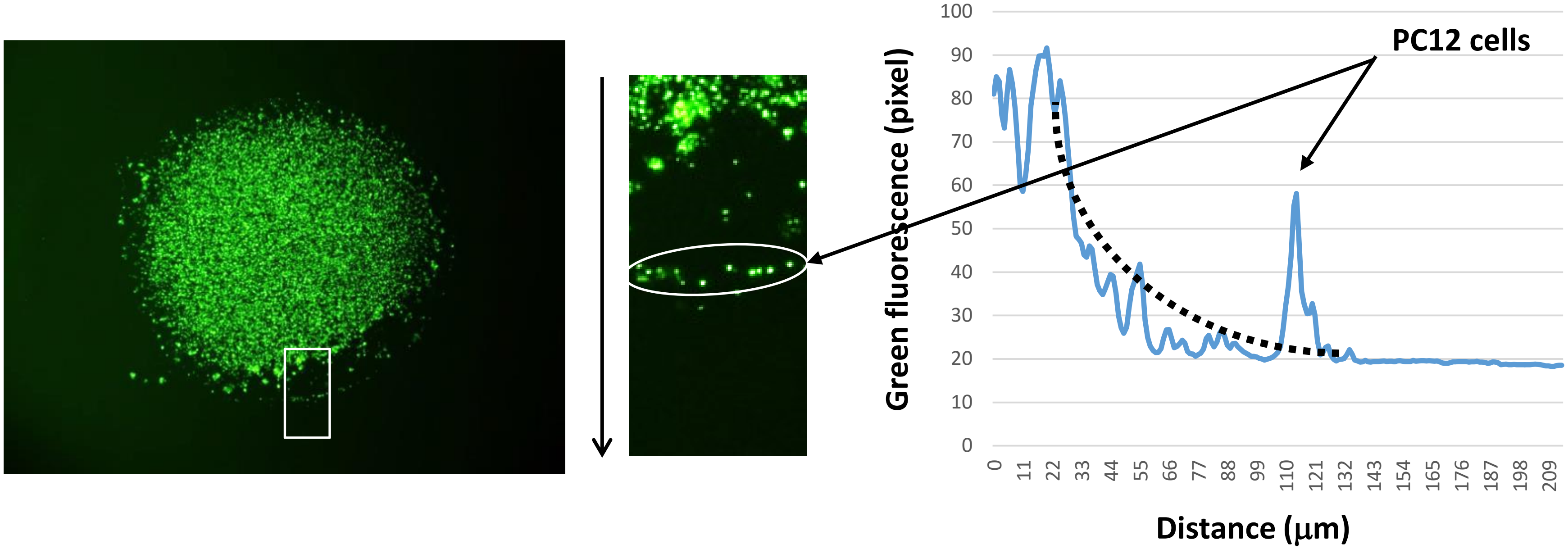

Supplementary Figure S4. Gradient of the encapsulated proteins released from PODS nanoparticle . Both pEGFP and pfNGF were mixed and spotted. PC12 cells were seeded and incubated for 5 days (left). Green fluorescence was scanned within the white box (middle) and measured by counting the number of pixels that exhibited green fluorescence (right). The aligned PC12 cells were detected by the fluorescence from PODS nanocrystal field. The dotted line indicates the extent of the EGFP gradient.
