# Supplementary material for: Sustained Neurotrophin Release from Protein Nanoparticles Mediated by Matrix Metalloproteinases Induces the Alignment and Differentiation of Nerve Cells
Source: Biomolecules. 2019 Sep 20;9(10):510. doi: 10.3390/biom9100510 (PMC6843502; doi:10.3390/biom9100510)
Supplement: Supplementary file 1 [file biomolecules-09-00510-s001.zip › Supplementary Data/Supplementary Table S1.pdf]

Supplementary Table S1. Fluorescence of EGFP released from crystals after incubation with MMPs

	Mean	SD
MMP-1	0.012	0.001
Mock	0.008	0.002
MMP-2	0.232	0.048
Mock	0.013	0.002
MMP-3	0.007	0.001
Mock	0.030	0.012
MMP-7	0.014	0.003
Mock	0.005	0.002
MMP-8	0.244	0.020
Mock	0.006	0.005
MMP-9	0.010	0.000
Mock	0.005	0.000
MMP-12	0.004	0.002
Mock	0.002	0.001

After pEGFP was incubated with each MMP for 72 hr, EGFP released from crystals was measured. Mock was incubated without MMPs.
