# Supplementary material for: Sustained Neurotrophin Release from Protein Nanoparticles Mediated by Matrix Metalloproteinases Induces the Alignment and Differentiation of Nerve Cells
Source: Biomolecules. 2019 Sep 20;9(10):510. doi: 10.3390/biom9100510 (PMC6843502; doi:10.3390/biom9100510)
Supplement: Supplementary file 1 [file biomolecules-09-00510-s001.zip › Supplementary Data/Supplementary Table S2.pdf]

Supplementary Table S2. Fluorescence of soluble EGFP after incubation with MMPs

	Mean	SD
MMP-1	4.58	0.02
Mock	4.28	0.01
MMP-2	1.81	0.02
Mock	2.29	0.10
MMP-3	0.78	0.02
Mock	2.27	0.21
MMP-7	0.18	0.00
Mock	3.34	0.02
MMP-8	1.07	0.03
Mock	1.07	0.00
MMP-9	4.14	0.13
Mock	4.27	0.21
MMP-12	5.12	0.23
Mock	5.22	0.12

After soluble EGFP was incubated with each MMP for 72 hr, fluorescence was measured. Mock was incubated without MMPs.
