# Supplementary material for: Sustained Neurotrophin Release from Protein Nanoparticles Mediated by Matrix Metalloproteinases Induces the Alignment and Differentiation of Nerve Cells
Source: Biomolecules. 2019 Sep 20;9(10):510. doi: 10.3390/biom9100510 (PMC6843502; doi:10.3390/biom9100510)
Supplement: Supplementary file 1 [file biomolecules-09-00510-s001.zip › Supplementary Data/Supplementary Table S3.pdf]

Supplementary Table S3. Fluorescence of EGFP after incubation with trypsin and chymotrypsin.

Enzyme	Fluorescence intensity (arbitrary units)
Trypsin	0.12
Chymotrypsin	0.15
No enzyme	0.03

After H1-tagged pEGFP (3 x 10^6 crystals/100 μl) was incubated with each 2 μg of trypsin and chymotrypsin for 12 hr at 37° C, the fluorescence of EGFP released from crystals was measured.
